# Rice *UCL8*, a plantacyanin gene targeted by miR408, regulates fertility by controlling pollen tube germination and growth

**DOI:** 10.1186/s12284-018-0253-y

**Published:** 2018-11-19

**Authors:** Fan Zhang, Yu-Chan Zhang, Jin-Ping Zhang, Yang Yu, Yan-Fei Zhou, Yan-Zhao Feng, Yu-Wei Yang, Meng-Qi Lei, Huang He, Jian-Pin Lian, Yue-Qin Chen

**Affiliations:** Guangdong Provincial Key Laboratory of Plant Resources, State Key Laboratory for Biocontrol, School of Life Sciences, Sun Yat-Sen University, Guangzhou, 510275 People’s Republic of China

## Abstract

**Background:**

Pollen tube formation and growth are crucial steps that lead to seed production. Despite the importance of pollen tube growth, the molecular mechanisms implicated in its spatial and temporal control are not fully known. In this study, we found an uclacyanin gene, *OsUCL8*, that regulates pollen intine deposition and pollen tube growth.

**Findings:**

The overexpression of *OsUCL8* led to a striking irregularity in pollen tube growth and pollination and thus affected the seed setting rate in rice; many pollen tubes appeared to lose the ability to grow directly into the style. Conversely, plants with *OsUCL8* knocked out and plants overexpressing *miR408*, a negative regulator of *OsUCL8*, had vigorous pollens with a higher germination rate. We further demonstrated that OsUCL8 mainly affects pollen intine formation. The addition of Vitamin B1 (VB1) significantly contributed to the germination of OXUCL8 pollen grains, suggesting that *OsUCL8* could be associated with VB1 production. Using a yeast two-hybrid system, we revealed that OsUCL8 interacts with the protein OsPKIWI, a homolog of the Arabidopsis FNRL protein. We thus hypothesized that OsUCL8 might regulate the production of VB components by interacting with OsPKIWI. This study revealed a novel molecular mechanism of pollen tube growth regulation.

**Conclusions:**

The rice plantacyanin family member OsUCL8 plays an important role in pollen tube formation and growth and, in turn, regulates fertility and the seed setting rate.

**Electronic supplementary material:**

The online version of this article (10.1186/s12284-018-0253-y) contains supplementary material, which is available to authorized users.

## Findings

Double fertilization is a crucial step in flowering plant reproduction. Highly orchestrated pollen-pistil interactions and signaling events enable plant species to avoid inbreeding and outcrossing with other species. This process starts with the adhesion of pollen to the stigma, followed by grain hydration, tube generation, stigma penetration, and tube elongation inside the style (Berger et al., 2008). Pollen grains induce the formation of pollen tubes after germination on the stigma and begin the long journey to deliver their sperm cells. Despite the importance of this crucial step in seed production, the molecular mechanisms implicated in the spatial and temporal control of pollen tube growth are not fully known (Johnson and Lord, 2006; Palanivelu and Tsukamoto, 2012). Plantacyanins belong to a subfamily of blue copper proteins (Ryden and Hunt, 1993) and have been proposed to be involved in the oxidative burst that occurs during pathogen infection and in the cross-linking and solubilization of cell wall materials (Nersissian et al., 1998). Arabidopsis plantacyanins can regulate reproduction by affecting anther development and pollination(Dong et al., 2005). Recently, we also showed that a rice plantacyanin gene, *OsUCL8*(*Oryza sativa* Uclacyanin like protein 8), could regulate grain yield and photosynthesis(Zhang et al., 2017). Further studies have revealed that the cleavage of *OsUCL8* by *miR408* affects copper homeostasis in the plant cell, which in turn affects the abundance of plastocyanin proteins and photosynthesis. These studies suggest that the plantacyanin family can have multiple functions during plant development. We also found that plants overexpressing *OsUCL8* showed semi-dwarf phenotypes, and the effective grains per panicle were dramatically lower than in WT plants. In crops, the seed setting rate is controlled by pistil and stamen development, including pollination. Thus, we asked whether *OsUCL8* also participates in pollination or sexual-reproduction-associated processes.

**Fig. 1:**
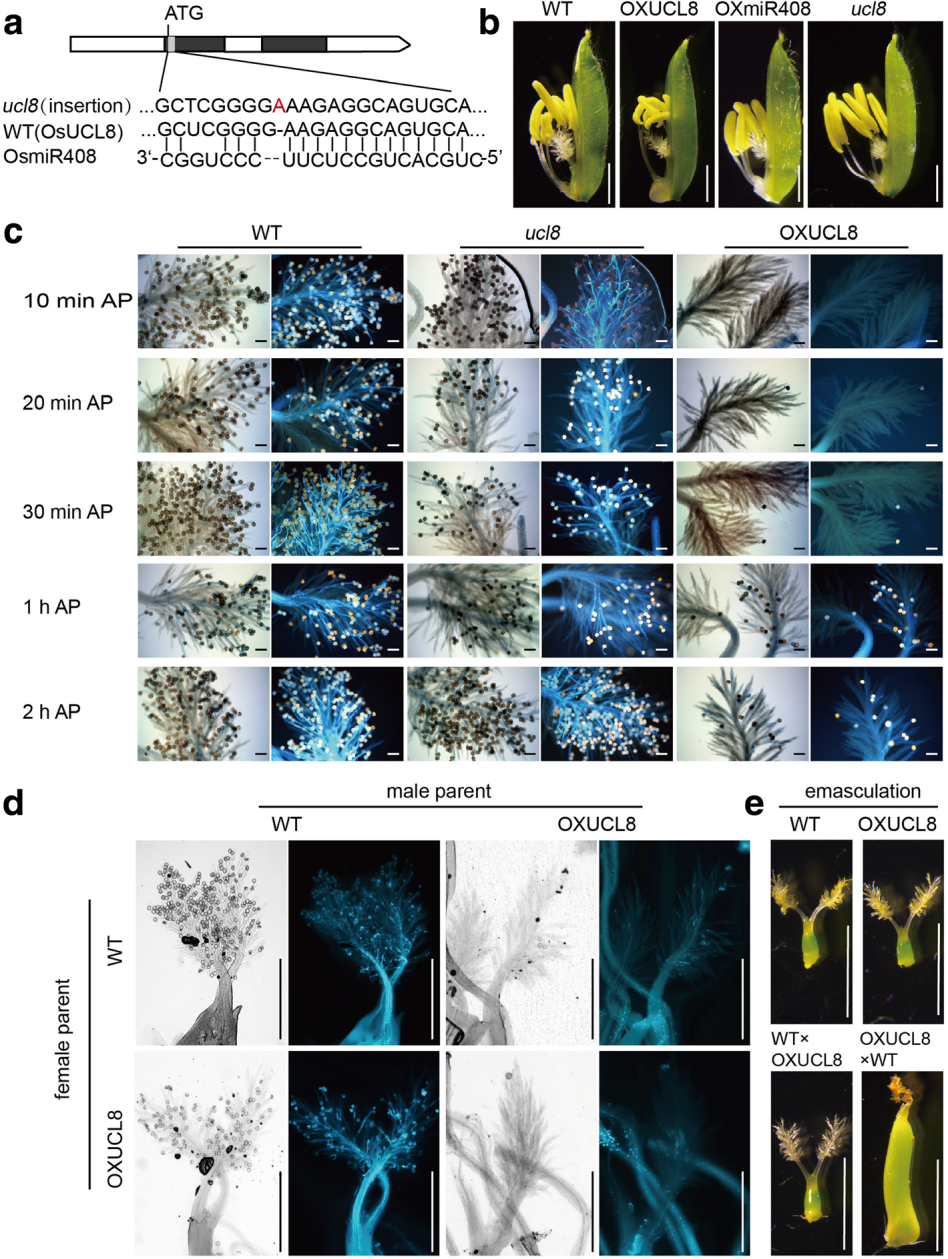
*OsUCL8* affected pollination and pollen germination. (**a**) Genomic structure of rice *OsUCL8* and constructions of the transgenic plants. (**b**) Mature spikelet of WT and transgenic plants. Scale bars, 1 mm. (**c**) Aniline blue staining of pollen tube growth in WT and mutants at 10 min, 20 min, 30 min, 1 h, 2 h after pollination (AP). Scale bars, 100 μm. (**d**) Reciprocal crosses between OXUCL8 and WT 2hs after artificial pollination, Scale bars, 500 μm. (**e**) Reciprocal crosses between OXUCL8 and WT 3 d after pollination, Scale bars, 3 mm

To demonstrate how OsUCL8 affects the seed setting rate, we used three *OsUCL8* mutants, including transgenic plants that ectopically expressed *OsUCL8* (OXUCL8), transgenic plants with *OsUCL8* knocked out via CRISPR-Cas9 (*ucl8*), and transgenic plants that ectopically expressed a microRNA, *miR408*, which has been shown to negatively regulate *OsUCL8*. Figure 1a showed the genomic structure of rice *OsUCL8*. We observed the floret structure and pollen fertility of the plants mentioned above. As shown in Fig. 1b and Additional file 1: Figure S1, the floret structures of three *OsUCL8* mutants are similar to those of the WT; only the stigmas of *OsUCL8*-overexpressing plants appear slightly smaller than those of wild-type, and the OXUCL8 plants have twisted anthers. We also observed sporogenesis and the mature pollen; no obvious differences were found between the three mutants and the WT from the meiosis stage to mature microspore development (Additional file 1: Figure S2), suggesting that *OsUCL8* did not affect male gametogenesis. To clarify the cause of the observed semisterility, mature embryo sacs were examined using whole-mount confocal laser scanning microscopy (WE-CLSM) of eosin B staining (Zeng et al., 2007). The results showed that almost all of the mature mutant embryo sacs were of the typical Polygonum type and had complete inner components with apparent polarity (Additional file 1: Figure S1c), suggesting that the mature mutant embryo sacs are functional and that the low grain setting rate of OXUCL8 may not result from a defect in female gametogenesis. In crops, the seed setting rate is controlled by pistil and stamen development, as well as pollination. Because neither male nor female gametogenesis showed defects, we speculated that the cause of the sterility in OXUCL8 plants might be associated with pollination or other sexual-reproduction-associated processes.

To validate this hypothesis, we performed in vivo pollen grain germination experiments using aniline blue staining(Kho et al., 1968) to detect pollen tube formation**.** The results showed that in WT and ucl8, the pollen grains attached to the stigmas and generated pollen tubes 10 min after pollination (AP); then, the pollen tubes entered the stigmas and grew rapidly 20 min AP (Fig. 1c). However, in OXUCL8, fewer pollen grains were observed on the stigmas at 20 min AP, and few pollen tubes were generated until 30 min AP. One hour AP, pollen tubes in the WT and *ucl8* plants easily entered the style tract and grew rapidly, whereas pollen tubes in the OXUCL8 mutants appeared to experience difficulty entering the style tract and grew extremely slowly. In the WT, several pollen tubes reached the ovary at 2 h AP; however, pollen tubes in the OXUCL8 mutant were abnormally delayed in the style tract. Consequently, no pollen tubes were found entering the embryo sac in the mutant, even at 2 h AP (Additional file 1: Figure S3).

To further confirm that the decreased seed setting rate of OXUCL8 plants was caused by abnormal germination of pollen grains on stigma in OXUCL8 plants, we then crossed the OXUCL8 and WT plants (the OXUCL8 plants served as male parent or female parent respectively). The female parent were first treated by hot water (42 °C, 5 min) to emasculation, then the pollens from the male parent were placed on the stigma of emasculated flower to bring about fertilization. We found that lots of pollen grains fixed to the stigma of both WT and OXUCL8 plants 2 h after artificial pollination when using WT as male parent. In contrast, when we placed OXUCL8 pollens on the WT stigma, there were only few pollen grains fixed to the stigma 2 h after artificial pollination (Fig. 1d). The cross success rate when using OXULC8 as female parent is 2.5 folds higher than that using OXUCL8 as male parent (Fig. 1e). These results indicate that the stigmas of OXUCL8 plants are ready for pollination, but the pollen grains of OXUCL8 failed to germinate on the stigma, thus the cause of the sterility in OXUCL8 plants is associated with abnormal pollen grain germination..

**Fig. 2:**
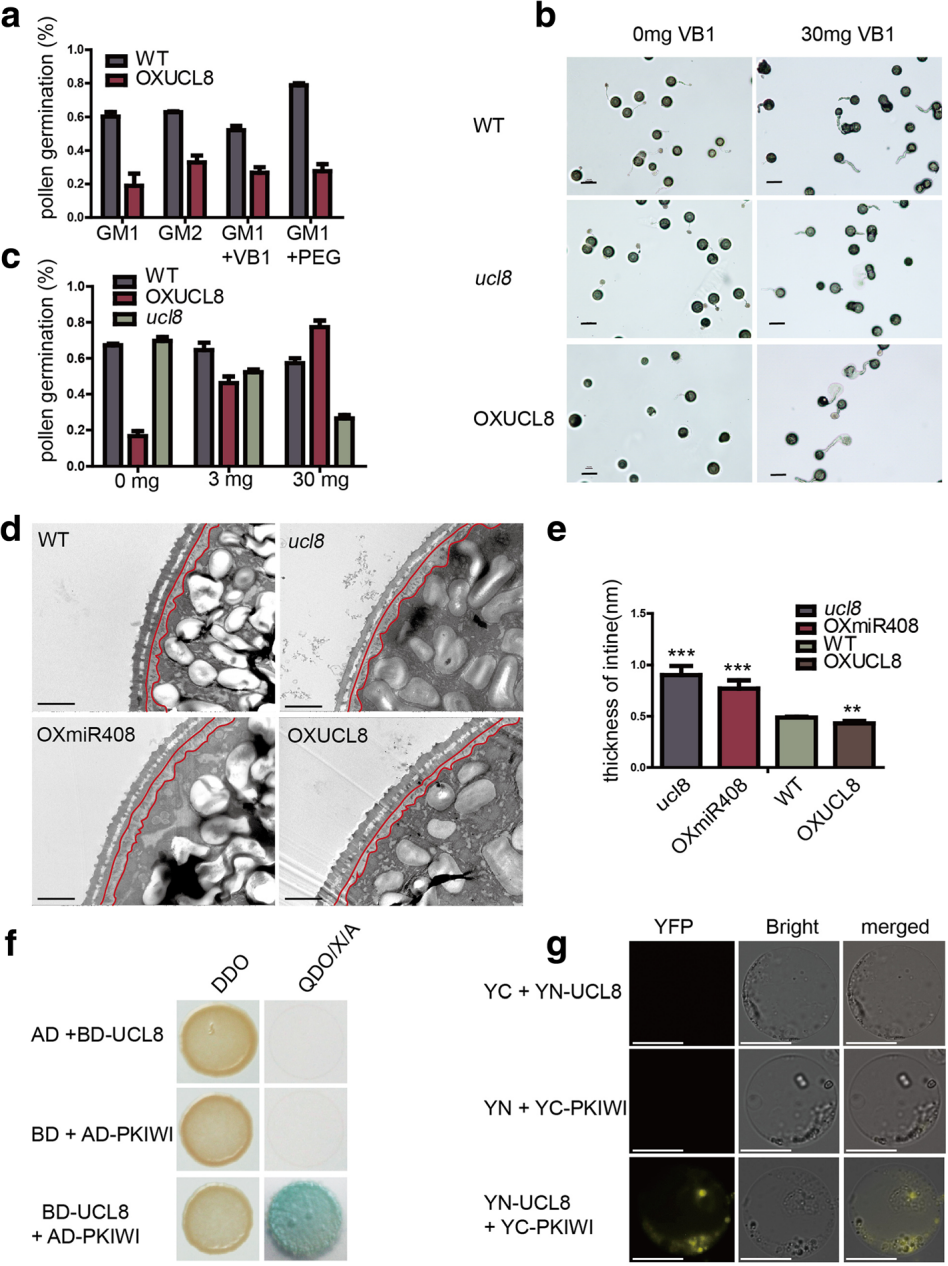
OsUCL8 regulates pollen intine and interacts with OsPKIWI protein. (**a**) Pollen germination rate of WT and mutants in different germination mediums,values are the means ± s.d. (*n* = 480 pollens from 3 plants). (**b**) Pollen germination of WT, *ucl8* and OXUCL8 pollens in GM1 with 0 or 30 mg/L VB1. Scale bars, 100 μm. (**c**) Pollen germination rate of WT, *ucl8* and OXUCL8 pollens in GM1 with 0, 3 and 30 mg/L VB1, values are the means ± s.d. (*n* = 480 pollens from 3 plants). (**d**) Observation of the ultrastructure of pollen wall for WT, OXUCL8, *ucl8* and OXmiR408 by TEM. The parts surrounded by red lines are intine, Scale bars, 2 μm. (**e**) The statistical results of intine thinkness, values are the means ± s.d. (*n* ≧ 77 pollens). (**f**) Detection of OsUCL8-OsPKIWI interaction with a yeast two-hybrid assay. The combinations of AD/BD-OsUCL8 and AD-OsPKIWI/BD were used as negative controls. (**g**) Verification of the interaction between OsUCL8 and OsPKIWI by BiFC assay in rice protoplasts. Empty YC and YN were used as negative controls. Scale bars, 10 μm. Asterisks (***) indicate *P* value < 0.0001 (t tests), Asterisks (**) indicate P value < 0.01 (t tests)

We then monitored the pollen tube germination process in vitro in two types of media. In the optimal GM1 (Mizuta et al., 2010), 60.3% of wild-type pollen grains germinated after 12 h of culture (Fig. 2a). By contrast, the percentage of germinated OXUCL8 pollen grains was drastically lower; only 19.0% of the pollen grains had germinated after 12 h of culture. In GM2 (Dai et al., 2007), 62.9% of wild-type pollen grains had germinated after 12 h of culture. By contrast, only 32.9% of OXUCL8 pollen grains had germinated after 12 h. Interestingly, the germination rate of OXUCL8 pollen grains was higher in liquid medium containing only extra PEG and VB1 than on solid medium. To determine whether PEG or VB1 affect the germination rate of OXUCL8 pollen, the same concentration of PEG or VB1 was added to the solid GM. In the +PEG group, 78.7% of the WT pollen germinated, and OXUCL8 germination improved to 27.6%. However, in the +VB1 group, the WT pollen germination dropped to 52%. This is possibly the reason that VB1 could induced the higher levels of reactive oxygen species as reported(Ahn et al., 2006; Maksimov et al., 2018). By contrast, the pollen germination of OXUCL8 still improved to 26.7% at a low concentration of VB1 (Fig. 2a), and very notably, the OXUCL8 pollen germination increased from 26.7% to 77.2% when the VB1 in the solid GM was increased from 0.3 mg/L to 30 mg/L (Fig. 2b and c), indicating that the higher VB1 concentration significantly contributed to the germination of OXUCL8 pollen grains. For comparison, we also investigated *ucl8*. The results clearly showed *ucl8* phenotypes are negatively correlated with those of OXUCL8, further supporting the observation that *OsUCL8* plays a regulatory role in pollen germination. The in vitro experiment also suggested that *OsUCL8* might affect the VB1 composition.

Pollen tubes can be identified based on the intine, which is the inside structure of the pollen wall(Edlund et al., 2004). Thus, we observed pollen wall structure using transmission electron microscopy (TEM) (Liu et al., 2013). The ultrastructure of the pollen walls of the WT, OXUCL8, *ucl8* and OXmiR408 plants was investigated. All plants developed an intact exine, but the intine was thinner in OXUCL8 pollen and thicker in the OXmiR408 pollen than in WT pollen (Fig. 2d and e), indicating that *OsUCL8* regulates pollen intine formation and affects pollen tube elongation in the style, consequently affecting fertility. To dissect the mechanism by which *OsUCL8* regulates pollen germination and pollen tube growth in rice, yeast two-hybrid (Y2H) screening was performed to identify OsUCL8-interacting proteins from a rice panicle cDNA Y2H library. After several screens, several proteins were identified (Additional file 1: Table S1). A fruit protein, PKIWI502 (Os02g0328300), was further validated using the full-length coding sequence of the PKIWI502 protein. The results indicated that OsPKIWI indeed interacted with OsUCL8 (Fig. 2f and g).

OsPKIWI is a homolog of the Arabidopsis FNRL protein (AT1G15140). FNR is a flavoenzyme that catalyzes the last step in linear photosynthetic electron transfer and produces the coenzyme NADPH (Koskela et al.,^2018^). The coenzyme NADPH has been reported to be involved in cell wall integrity and growth at the pollen tube tip (Boisson-Dernier et al., 2013; Potocky et al., 2007). Very interestingly, VB1 is also an essential coenzyme for carbohydrate metabolism and is involved in energy generation(Bazurto et al., 2015). We have found that the high VB1 concentrations significantly contributed to the germination of OXUCL8 pollen grains (Fig. 2a), suggesting that the germination defect in OsUCL8-overexpressing pollen grains might be related to the components of the VB1 sink in the intine wall. We hypothesized that OsUCL8 might regulate the production of VB1 components by interacting with OsPKIWI. This hypothesis deserved to be further validated.

In conclusion, we discovered an uclacyanin gene *OsUCL8* which regulates pollen germination and pollen tube growth, and in turn regulates fertility and seed setting rate. We further demonstrated that *OsUCL8* mainly affects pollen intine formation. Addition of VB1 could significantly contribute to the germination of OXUCL8 pollen grains, suggesting that VB1 could be the important component of pollen intine. We revealed that OsUCL8 interacts with OsPKIWI, the homolog of Arabdopsis FNRL protein. We thus hypothesized that OsUCL8 might regulate the production of VB1 components through interaction with OsPKIWI. The study revealed a novel molecular mechanism in regulating pollen tube growth.

## Additional file

Additional file 1: Materials and Methods are presented in supplemental files (Ma et al., 2015, Ross et al., 1996). (DOCX 2745 kb)

## Abbreviations

AP: After pollination
DAPI: 4′,6-diamidino-2-phenylindole
GM: Germination mediums
TEM: Transmission electron microscopy
VB1: Vitamin B1
WE-CLSM: Whole-mount eosin B staining confocal laser scanning microscopy
WT: Wild type
Y2H: Yeast two-hybrid
