# Supplementary material for: Rice UCL8, a plantacyanin gene targeted by miR408, regulates fertility by controlling pollen tube germination and growth
Source: Rice (N Y). 2018 Nov 19;11:60. doi: 10.1186/s12284-018-0253-y (PMC6242803; doi:10.1186/s12284-018-0253-y)
Supplement: Supplementary file 1 — Materials and Methods are presented in supplemental files (Ma et al., 2015, Ross et al., 1996). (DOCX 2745 kb) [file 12284_2018_253_MOESM1_ESM.docx]

**Supplemental materials for “Rice UCL8, a plantacyanin gene targeted by miR408, regulates fertility by controlling pollen tube germination and growth”**

**Materials and methods**

**Plant growth conditions and generation of transgenic rice plants**

The Zhonghua 11 (Oryza sativa japonica) rice cultivar was used in the experiments. Rice plants were grown in the field in Guangzhou, China. The constructions of OXmiR408 and OXUCL8 mutantshave been described previously (Zhang et al., 2017). The ucl8 mutants were generated using CRISPR-Cas9-based genome editing technology (Ma et al., 2015). The primers used were as follows: 5’-GCCAGCATCGATGGCTCGGGGAAG-3 and 5’- AAACCTTCCCCGAGCCATC

GATGC-3’.

**DAPI staining**

The DAPI staining was as described previously (Ross et al., 1996). Young panicles were fixed with Carnoy’s solution (ethanol:glacial acetic, 3:1). Anthers at the proper stages were washed twice with water, and then placed in a small drop of 60% acetic acid to release microspore mother cells. Slides with microspore mother cells were stained in DAPI solution (1 µg/mL DAPI in a buffer with 50% glycerol and 10 mM citrate, pH 4.5), covered with a cover glass and examined under a fluorescence microscope (Leica DM5000B).

**Eosin B staining**

The eosin B staining for embryo was performed as previously described (Zeng et al., 2007). The ovaries of WT and mutants were dissected in 70% ethanol under a binocular dissecting microscope, and sequentially hydrated in 50%, 30% ethanol and water. The hydrated ovaries were pretreated in 2% aluminium potassium sulphate for 20 min then stained with 10 mg/l of eosin B solution for 10-12 h at room temperature. Subsequently, the samples were post-treated in 2% aluminium potassium sulphate for 20 min and then rinsed three times with water, followed be dehydration through an ethanol series (30%, 50%, 70%, 90% and 100%). Finally, the samples were transferred to a mixture of absolute ethanol and methyl salicylate (1:1) for 1 h and then soaked in pure methyl salicylate solution for at least 1 h. The samples were examined under a confocal laser scanning microscope (Zeiss 7 DUO NLO).

**Aniline blue staining**

In vivo pollen grain germination experiments have been described previously（Kho, et al., 1968）. Pistils were collected at 10 min, 20 min, 30 min, 1 h, 2 h and 12 h after pollination and fixed overnight in Carnoy’s solution (ethanol:glacial acetic, 3:1) at room temperature. The fixed pistils were softened by maceration with 1 N NaOH for 30 min at 60°C. After being thoroughly washed in water the pistils then stained with 0. 1% aniline blue dissolved in 0.1 M K_3_PO_4_for 5-10 min or as long as 10 h. Transfer to a slide, and observed by a microscope with UV light. At least 30 pistils were observed.

**In Vitro Germination of Pollen Grains**

Mature pollen grains were collected by shaking panicles gently during anthesis. These collected fresh pollens were transferred to germination mediums (GM1: 1 mg H_3_BO_3_, 3 mg Ca(NO_3_)_2_, 1.7 g sucrose and 0.07 g agarose in 10 mL sterilized water; GM2：20% sucrose, 10% PEG 4000, 3 mM Ca(NO_3_)_2_, 40 mg/L H_3_BO_3_, 3 mg/L vitamin B1) and cultured at 28-30 °C. Germinated pollen grains were examined under a microscope. We calculated the germination rate of each field，and more than 12 fields （40-50 pollens/field）from 3 plants were counted by Image J.

**TEM for Pollen Ultrastructural Analyses**

Anthers from WT and mutants, were fixed with 3% glutaraldehyde and 2% osmic acid, dehydrated, and embedded in Spurr’s resin (Sigma-Aldrich). Ultrathin sections were stained with 4% uranylacetate, then observed by a transmission electron microscope (JEM-1230; JEOL). The thickness of the intine was determined at three different regions of each pollen, and in each region we measured the maximum and the minimum thickness by Image J. More than 77 pollen grains were used for intine thickness analysis for each sample.

**Yeast Two-Hybrid Analysis**

The yeast two-hybrid assay was performed using a Matchmaker Gold Yeast Two-Hybrid System (Clontech). The OsUCL8 and OsPKIWI ORF was fused in frame to pGBKT7 and pGADT7, respectively, to construct BD-UCL8 and AD-PKIWI. Both constructs were used to transform to Y2HGold Yeast Strain. The primers used were as follows: UCL8-F: 5’-CGGAATTCGCCACCTACTACGTCG-3’; UCL8-R: 5’-CGGGATCCTCACACGGCGGTGACGA-3’; PKIWI-F: 5’-CGGAATTCATGGC TGCGGTCGCCAA-3’; PKIWI-R: 5’-CGGGATCCCTAATAGTTGGTCAAGAT TT-3’.

**BiFC Assays in Rice Protoplasts**

OsUCL8 and OsPKIWI cDNAs were cloned into the pUC-YN and pUC-YC vector, respectively. The amplified primers of OsUCL8 were 5'-TCTAGAA TGGCTCGGGGAAGAGGC-3', and 5'-GGTACCCACGGCGGTGACGACCA-3', and The amplified primers of OsPKIWI were 5'-TCTAGAATGG CTGCGGTCGCCAA-3' and 5'-CTCGAGATAGTTGGTCAAGATTTTATCTGGT GATACA-3'. To isolate protoplasts, 14d old rice shoots were cut into approximately 0.5-mm strips using sharp razors. Then the strips were incubated in an enzyme solution (1.5% cellulose RS, 0.75% macerozyme R-10, 10 mM MES (pH 5.7), 0.6 M mannitol, 10 mM CaCl2 and 0.1% BSA) for 3-5 h in dark with gentle shaking (40 rpm). After enzymatic digestion, enzyme solutions were filtered through 40-μm nylon mesh into round bottom tubes. Protoplasts were collected by centrifugation at 100 g for 5 min. After washing once with W5 solution (154 mM NaCl, 125 mM CaCl2, 5 mM KCl and 2 mM MES), the pellets were then re-suspended in MMG solution (15 mM MgCl2 and 4 mM MES, 0.4 M mannitol) at a concentration of 2×10^6^ cells mL^-1^. 20 µl (10-20 µg ) plasmid was used to transformation of 200 µl protoplasts. Mixed the plasmid and protoplasts in a 2 ml tube with 220 µl PEG solution (40% PEG4000 (W/V)，0.4 M mannitol，100 mM CaCl2) in the tube. After gently mixed, stay the tube in room temperature for 30 min, and wash once by 880 µl W5 solutions. Then suspened the transformed protoplasts with 1 ml W5 solution. After incubation at 28-30 °C for 14 h, fluorescence images for yellow fluorescent protein (YFP) were obtained using a confocal laser-scanning microscope (Zeiss 7 DUO NLO).


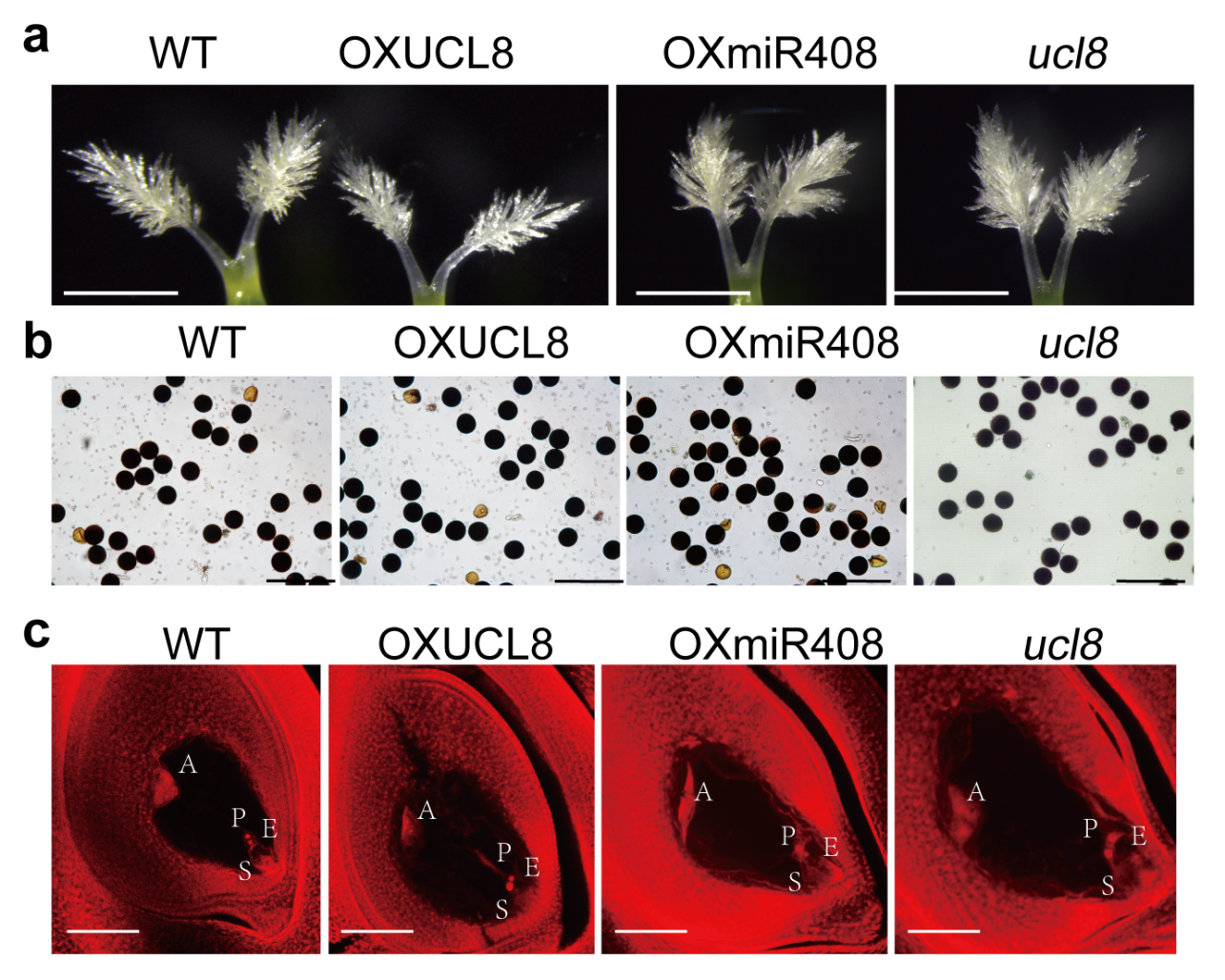


Figure S1. Gross morphology of WT and mutants. (a) Stigma of WT and transgenic plants. Scale bars, 1 mm. (b) Pollen grains stained with I_2_-KI. Scale bars, 100 µm. (c) Mature embryo sacs of WT, *ucl8* , OXmiR408 and OXUCL8 plants by eosin B staining. Scale bars, 100 µm.


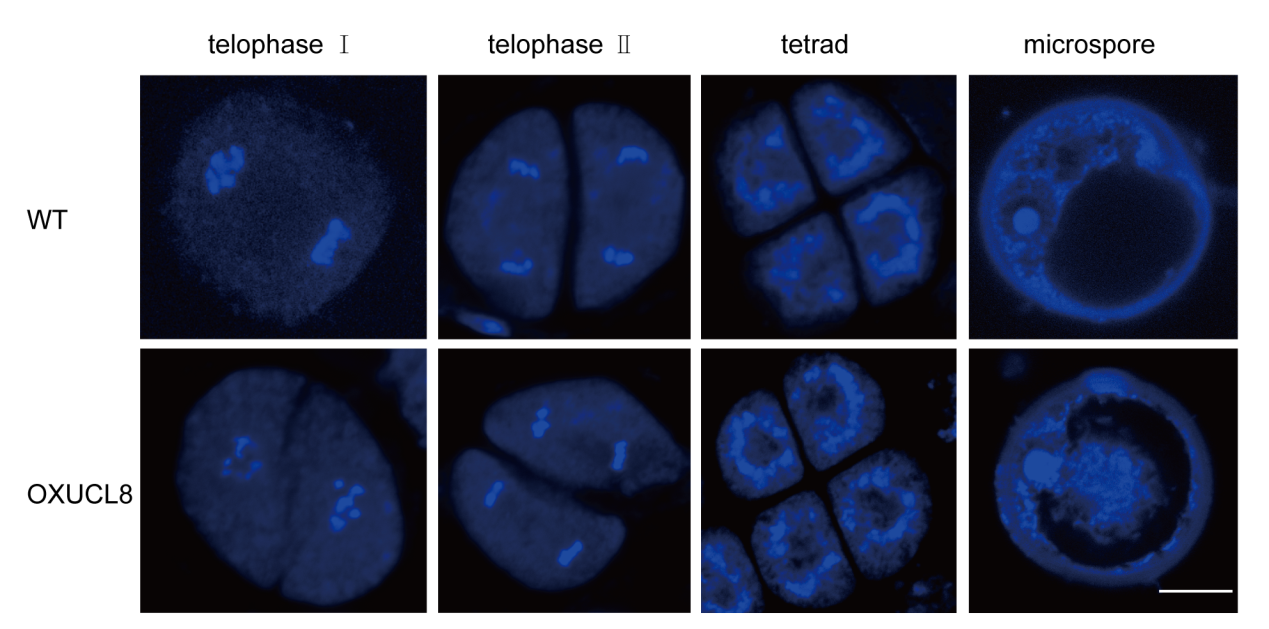


Figure S2 The meiosis processes and late micropore stage of WT and Ooxucl8. Scale bars, 10 µm.


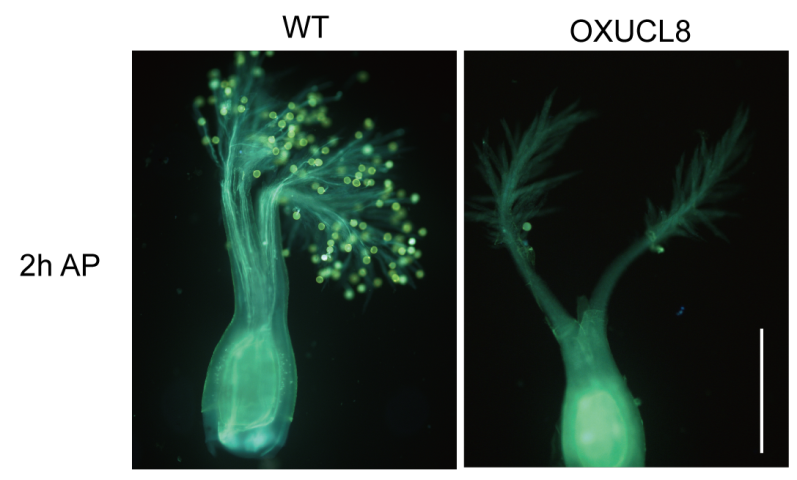


Figure S3 Pollen tubes of WT and OXUCL8 2 h AP. Scale bars, 1 mm.

| MSU ID | RAP ID | Gene Product Name |
| --- | --- | --- |
| LOC_Os11g29200 | Os11g0482100 | Similar to Protein transport protein Sec24-like CEF |
| LOC_Os06g01210 | Os06g0101600 | Plastocyanin |
| LOC_Os02g22260 | Os02g0328300 | fruit protein PKIWI502 |
| LOC_Os05g04380 | Os05g0134400 | peroxidase precursor |
| LOC_Os04g32850 | Os04g0401000 | pi21 |
| LOC_Os08g33710 | Os08g0434100 | ribonuclease T2 family domain containing protein |
| LOC_Os02g33500 | Os02g0538000 | threonyl-tRNA synthetase |
| LOC_Os03g57220 | Os03g0786100 | hydroxyacid oxidase 1 |
| LOC_Os05g27780 | Os05g0344200 | expressed protein |
| LOC_Os02g35560 | Os02g0563800 | Cyclin-like F-box domain containing protein |
| LOC_Os09g27420 | Os09g0446800 | formate-tetrahydrofolate ligase activity,ATP binding |
| LOC_Os03g55930 | Os03g0769100 | Similar to 9S ribosomal protein |
| LOC_Os04g23040 | Os04g0296700 | Non-protein coding transcript |

Table S1. Genes screened out by yeast two-hybrid screening
